# On the Origin of the Non‐Arrhenius Na‐ion Conductivity in Na_3_OBr

**DOI:** 10.1002/anie.202314444

**Published:** 2023-11-14

**Authors:** Brigita Darminto, Gregory J. Rees, John Cattermull, Kenjiro Hashi, Maria Diaz‐Lopez, Naoaki Kuwata, Stephen J. Turrell, Emily Milan, Yvonne Chart, Camilla Di Mino, Hyeon Jeong Lee, Andrew L. Goodwin, Mauro Pasta

**Affiliations:** ^1^ Department of Materials University of Oxford Oxford OX1 3PH United Kingdom; ^2^ The Faraday Institution Harwell Campus Oxford OX11 0RA United Kingdom; ^3^ Department of Chemistry University of Oxford Oxford OX1 3TA United Kingdom; ^4^ National Institute for Materials Science Tsukuba 305-0044 Japan; ^5^ Diamond Light Source Oxford OX11 0DE United Kingdom; ^6^ Department of Materials Science and Engineering Ulsan National Institute of Science and Technology Ulsan 44919 South Korea

**Keywords:** non-Arrhenius behaviour, poorly crystalline impurities, sodium antiperovskites, solid sodium-ion conductors, solid-state batteries

## Abstract

The sodium‐rich antiperovskites (NaRAPs) with composition Na_3_OB (B=Br, Cl, I, BH_4_, etc.) are a family of materials that has recently attracted great interest for application as solid electrolytes in sodium metal batteries. Non‐Arrhenius ionic conductivities have been reported for these materials, the origin of which is poorly understood. In this work, we combined temperature‐resolved bulk and local characterisation methods to gain an insight into the origin of this unusual behaviour using Na_3_OBr as a model system. We first excluded crystallographic disorder on the anion sites as the cause of the change in activation energy; then identified the presence of a poorly crystalline impurities, not detectable by XRD, and elucidated their effect on ionic conductivity. These findings improve understanding of the processing‐structure‐properties relationships pertaining to NaRAPs and highlight the need to determine these relationships in other materials systems, which will accelerate the development of high‐performance solid electrolytes.

## Graphical abstract

Poorly crystalline impurities consisting of Na_2_O, Na_2_O_2_, and NaOH were detected in Na_3_OBr antiperovskite synthesised with commercially available Na_2_O. These impurities form a eutectic mixture which melts at around 250 °C, reducing the activation energy for Na^+^ conduction from 0.47 eV to 0.43 eV.

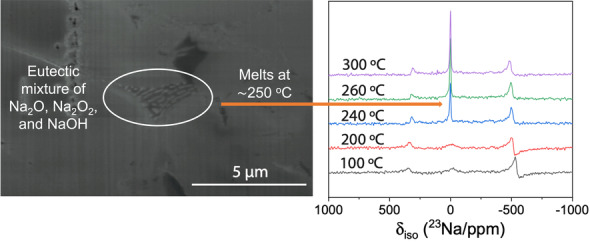

## Introduction

Sodium metal batteries (SMBs) have attracted significant attention as a potential alternative to lithium‐ion batteries (LiBs) due to their several key advantages: the abundance of sodium (1200 times more common than lithium), high theoretical specific capacity (1166 mA h g compared to 372 mA h g of graphite anodes in LiBs), and the low solubility of sodium in aluminium, enabling its use as a lightweight and inexpensive current collector.[^1^, ^2^, ^3^] Despite these benefits, combining a Na metal anode with an organic liquid electrolyte presents very serious safety risks. The high chemical activity of Na metal anodes can lead to unavoidable side reactions and uncontrolled dendrite growth, causing a continuous decline in electrochemical performance and potentially leading to internal short circuits, thermal runaway, and even fires.[^4^, ^5^, ^6^, ^7^, ^8^, ^9^] Solid‐state sodium metal batteries (SSMBs) have been proposed as alternatives. There has been an increasing interest in Na‐rich antiperovskite (NaRAP) materials as solid‐state electrolytes (SSEs) for SSMBs over the last decade owing to their stability against Na metal, low synthesis temperatures (<500 °C), and relatively soft mechanical properties (bulk modulus: ≈60 GPa) which facilitate processing conditions and improve contact with the active materials.[^10^, ^11^, ^12^, ^13^, ^14^] Their general formula is Na_3_AB, where A is a divalent anion and B is either a monovalent or a cluster anion.

The room temperature Na‐ion conductivity varies widely depending on composition and processing conditions, with the highest value of 4.4×10^−3^ S cm^−1^ reported by Sun et al. for Na_3_O(BH_4_); however, attempts to reproduce their results have been unsuccessful.[^10^, ^11^, ^15^] Interestingly, non‐Arrhenius behaviour resulting in a decrease in activation energy for ion conduction above 250 °C has been observed across different antiperovskite compositions (Li, K and Na).[^16^, ^17^, ^18^, ^19^, ^20^, ^21^, ^22^, ^23^] The origin of this behaviour is not yet well understood. For antiperovskite compositions with cluster anions like Na_3_O(NO_2_) and Na_2_(NH_2_)(BH_4_), deviation from Arrhenius behaviour is often assigned to activation of the cluster anion rotations and correlated motion between these rotations and the translating cation.[^17^, ^18^]

For the compositions with single halide anions, such behaviour is usually attributed to structural instability, which includes phase transitions, order‐disorder transitions of the ion sublattice, and crystal melting.[^16^, ^24^, ^25^] Zheng et al. argued that the non‐Arrhenius behaviour observed in K_3_OI could be related to the effect of K vacancies on the ion kinetics of the system.^[16]^ K_3_OI stays crystalline up to 350 °C—well above the transition temperature of 240 °C. Through ab‐initio molecular dynamics (AIMD) simulations, the authors demonstrated that at high temperatures local anion disordering is activated, and K‐ions migrate not only via the K vacancies, but also through the relatively large vacancies around the disordered I−O sites, thus enhancing K‐ion transport.^[16]^ Zhang et al. reported similar behaviour as the main driver for enhanced Li ionic mobility in Li_3_OCl, and referred to the phenomenon as ‘sublattice melting’. To ensure the formation of energetically unstable Li_3_OBr, they performed in situ variable temperature neutron scattering experiments at high pressures (3.1 and 6.5 GPa). An abrupt volume increase was observed between 350 °C and 400 °C, which corresponds to ≈0.8 T_m_, in agreement with AIMD simulations reported by their group.[^24^, ^25^] On the other hand, Dawson et al. performed molecular dynamics (MD) calculations on Li_3_OB (B=Cl or Br) and Na_3_OB (B=Cl or Br) for fast‐conducting solid electrolytes with an alkali‐halide partial Schottky defect concentration of *δ*=0.038 and did not observe any deviations from Arrhenius behaviour in their ionic conductivities at elevated temperatures.^[26]^ The same behaviour is observed in Li_2_OHCl and suggested to be different from that reported for K_3_OI and Li_3_OBr. Wang et al. argued that the defects on the Li site and the movement of H around the O atom give rise to a change in the ionic conduction pathway as temperature increases.^[27]^ Despite the initial similarities, these varying reports on several antiperovskite families indicate that the origin of non‐Arrhenius behaviour is influenced by the type and concentration of defects present in the structure and is not necessarily the same across different families.

Na_3_OBr was chosen for this experiment as it is reported to show non‐Arrhenius behaviour, the origin of which is not fully understood.^[20]^ Furthermore, this composition is predicted to be stable in the cubic antiperovskite structure on the basis of its Goldschmidt tolerance factor, *t*, of 0.89. This is a dimensionless parameter calculated from the ratio of the ionic radii of the constituent ions in the material.[^28^, ^29^] A value of *t*=1 represents the perfect case for the formation of a cubic structure.^[28]^ Multiple cubic NaRAPs with *t* between 0.84 and 0.97 have been synthesised and the value for Na_3_OBr is within this range.^[15]^ We studied variable‐temperature X‐ray diffraction (XRD), variable‐temperature nuclear magnetic resonance (NMR), variable‐temperature electrochemical impedance spectroscopy (EIS), differential scanning calorimetry (DSC), and scanning electron microscopy (SEM). The combination of these techniques allowed us to gain new insights into this unusual behaviour.

## Results and Discussion

We synthesised Na_3_OBr with commercially available Na_2_O, which contains ≈80 % Na_2_O and ≈20 % Na_2_O_2_. The significant amount of Na_2_O_2_ was taken into account in the precursor ratio calculation, as seen in the equation below.

(1) $$0.8{\mathrm{Na}}_{2}O+0.2{\mathrm{Na}}_{2}O_{2}+\mathrm{NaBr}\to {\mathrm{Na}}_{3}\mathrm{OBr}+0.1O_{2}$$

**Figure 1:**
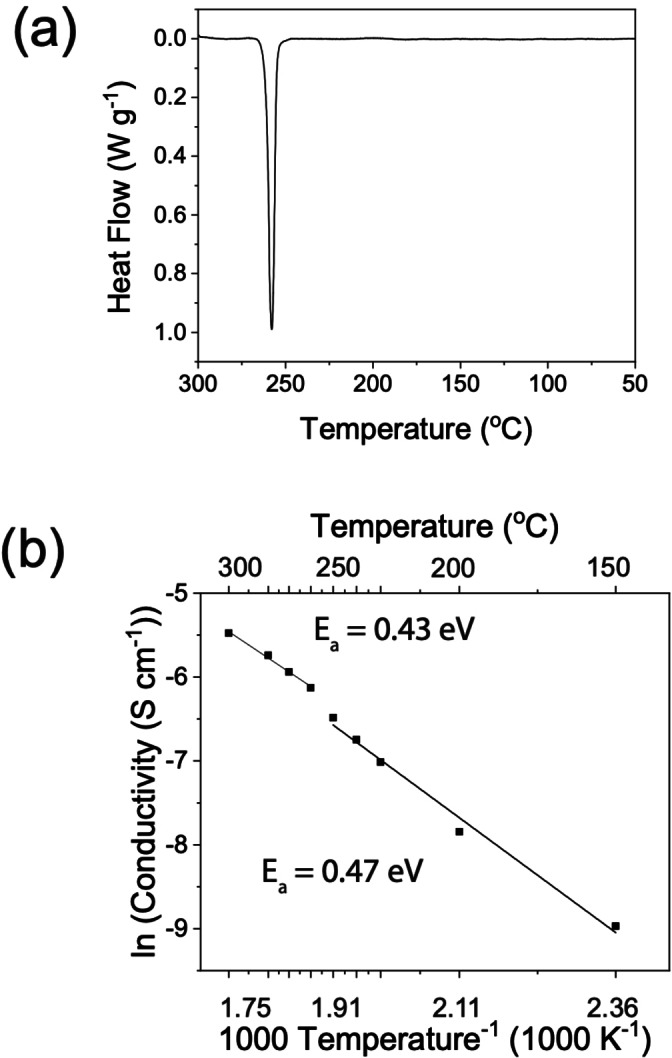
In‐house characterisations of as‐synthesised Na_3_OBr. (a) DSC of Na_3_OBr showing an endothermic peak with an onset at 247 °C. (b) Arrhenius plot of total ionic conductivity of Na_3_OBr with a step increase observed at 250 °C.

**Figure 2:**
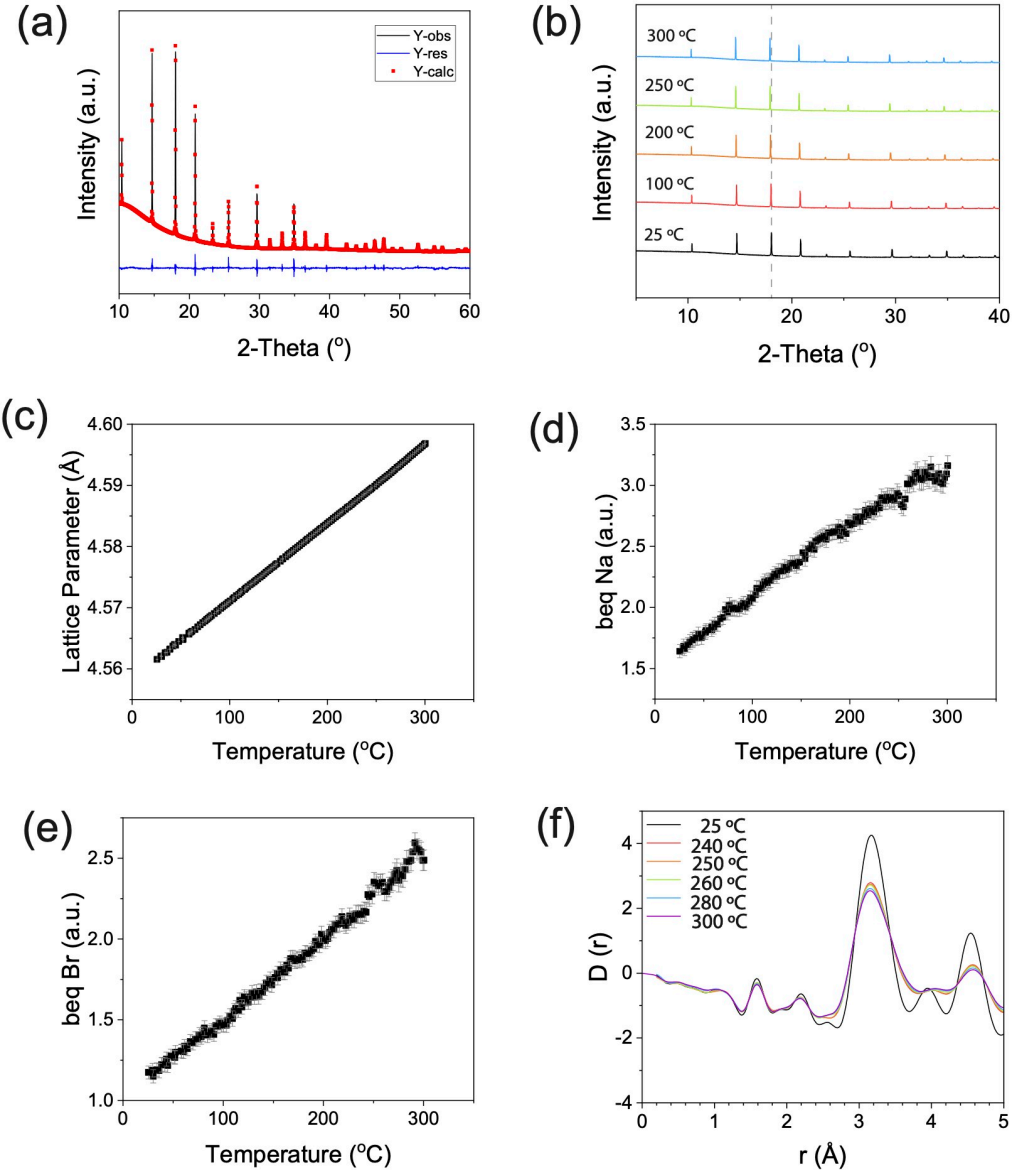
(a) Rietveld refinement of the room temperature synchrotron X‐ray diffractograms of synthesised Na_3_OBr with an X‐ray wavelength of 0.825 318 Å. The black line is the experimental data, the red line is the fit, and blue line is the difference between them. (b) Variable temperature experiment. Sequential Rietveld refinements were performed on the variable temperature data, and the changes in (c) lattice parameter, (d) thermal parameter of Na (*b_eq_* Na), and (e) thermal parameter of Br (*b_eq_* Br) of Na_3_OBr are plotted as a function of temperature. These show thermal expansion with no presence of crystalline deviation. (f) Differential correlation function *D(r)* calculated from experimental X‐ray total structure factor *F(Q)*. Measurement was done with an X‐ray wavelength of 0.161 669 Å.

A combination of high‐energy ball milling and subsequent heating is expected to decompose Na_2_O_2_ into Na_2_O and O_2_. This synthesis procedure has been reported multiple times in the literature and led to successful formation of NaRAPs.[^12^, ^17^] Differential scanning calorimetry (DSC) was performed to study the thermal behaviour of the Na_3_OBr, shown in Figure 1a. Upon heating, an endothermic peak was observed with an onset at 247 °C and maximum at 258 °C. Previous studies on Li, Na and K antiperovskites also showed similar endothermic peaks (K_3_OI: 245 °C, Li_2_OHBr: 254 °C, Na_3_OBr: 255 °C, and Na_3_OCl: 191 °C). Some studies attributed these peaks to melting of the antiperovskite phases and others to anion disordering.[^14^, ^16^, ^17^, ^19^] Except for this peak, no other peaks were observed in the DSC plot during heating to 300 °C. Additionally, the bulk material remains solid up to 300 °C, which is confirmed by variable temperature synchrotron XRD, Figure 2b, meaning that the peak does not correspond to the melting of Na_3_OBr.

Temperature‐dependent potentiostatic electrochemical impedance spectroscopy (PEIS) was used to investigate the effect of temperature on ionic conductivity. A pressure of 60–70 MPa was applied to the cell during the measurements to maintain good contact between gold blocking electrodes and the Na_3_OBr pellet. To determine the relative contributions of the electrolyte bulk and boundaries to the total impedance, the setup was modelled as an equivalent circuit shown in Figure S1. R_1_ and CPE_1_ correspond to bulk Na^+^ conduction while R_2_ and CPE_2_ correspond to conduction through the boundaries. The calculated total ionic conductivities are presented in Figure 1b. There is a step change in the total ionic conductivity at 250 °C, the temperature at which the endothermic peak was observed in the DSC, which corresponds to a reduction of activation energy from 0.47–0.43 eV. These activation energies are slightly lower than the reported values (0.52–1.14 eV), possibly due to the higher pressure applied to the cell during the measurements.[^10^, ^12^, ^20^, ^30^, ^31^] Refinements were performed over variable temperature synchrotron XRD data to elucidate the origin of this phenomenon.

In agreement with previous reports, the room temperature (RT) XRD pattern was fitted to the cubic space group $Pm\bar{3}m$ with a unit cell of *a*=4.562(6) Å, using a Rietveld refinement (*R_wp_* =1.02 %). No Bragg peaks other than those which belong to the Na_3_OBr are present in the diffraction pattern, thus no other crystalline species are present at significant concentrations in our sample. To understand how the crystal structure evolves with temperature, sequential Rietveld refinements were performed on the synchrotron XRD data between RT and 300 °C, with a step size of 2.5 °C (Figure 2b). Any sign of discontinuity in the lattice parameter and elemental thermal parameters (*b_eq_*) could give an indication of an order to disorder transition. The intensities of the Na_3_OBr peaks do not change drastically above 247 °C, hence the Na_3_OBr powder did not melt. The Bragg peak positions shift to lower angles due to thermal expansion of the Na_3_OBr lattice, but the rate of change in lattice parameter is constant with temperature, even above 267 °C where the endothermic peak ends, as seen in Figure 2c. When fitted with a straight line, the deviation of each point from the fit can be plotted (Figure S2). A change in the deviation trend above 247 °C was observed; however, the deviations are of the order of 10^−4^ Å, making them negligible compared to the size of any atom in the structure. Out of the three elements contained in our sample, we decided to focus our attention on the thermal parameters of Na and Br. This is because the thermal parameters of O have significant error bars (10–20 %) due to O being the weakest X‐ray scatterer of the three. The thermal parameter of Na (Figure 2d) was found to be higher than that of Br (Figure 2e), but in both cases the changes are only very small (≈1 %) around 250 °C. So, just as there is no evidence for abrupt behaviour in the lattice parameter variation, neither is there any sign in atomic displacements of discontinuous behaviour across the temperature range studied.

In order to investigate the local structure of Na_3_OBr, we looked at the pair distribution function (PDF) in the range of 0 Å to 5 Å (Figure 2f, full range is available in Figure S3). The majority of the peak positions shift to higher distances while the widths broadens as the temperature is increased. This behaviour can be attributed to thermal expansion and the thermal population of phonons. We note that an order‐disorder transition and a phase change (melting of Na_3_OBr) are absent in this temperature range. From this synchrotron XRD study, we can deduce that the endothermic peak in the DSC and the non‐Arrhenius behaviour of the ionic conductivity arise from neither the melting of Na_3_OBr nor an order‐disorder transition.

**Figure 3:**
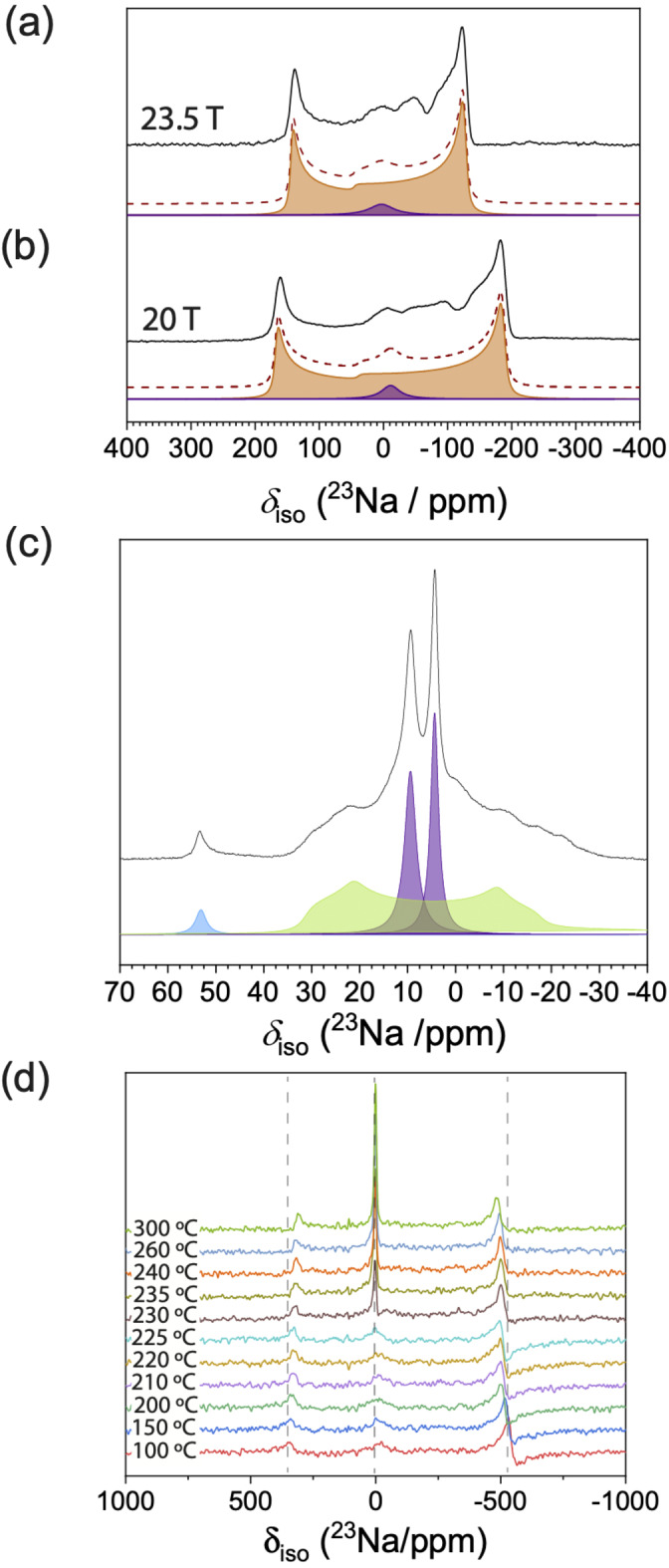
The room temperature ^23^Na static WURST‐echo NMR spectra (black) of Na_3_OBr at (a) 23.5 T (*ν*_0_=264.6 MHz) and (b) 20 T (*ν*_0_=224.9 MHz).The orange dotted lines represent the sum of the simulated Na_3_OBr (orange) and impurity resonances (purple). (c) High resolution MAS NMR (MAS, *ν_R_* =20 kHz) of the sample; impurity resonances were deconvoluted into three components: Na_2_O_2_ (purple), Na_2_O (blue), and NaOH (green). (d) The variable temperature ^23^Na static NMR spectra of Na_3_OBr (*ν*_0_=132.32 MHz).

Having excluded Na_3_OBr melting and order‐disorder transition as the potential origin of non‐Arrhenius behaviour, we probed the local Na environment by static ^23^Na NMR spectroscopy. The spectra in Figure 3a show two components, a broad (136 kHz at 11.7 T, 80 kHz at 23.5 T) quadrupolar peak and a narrower component at ≈0 ppm (shown in purple), which arises from impurities. The wideline Na_3_OBr (orange) is deconvoluted to give *δ_iso_* =44 ppm, *X_Q_* =11.9 MHz and *η_Q_* =0, which are similar to the reported values for Na_3_OCl.^[32]^ The large quadrupolar peak arises from the non‐symmetric octahedral coordination of Na ions with two O ions and four coplanar Br ions. Solid‐state ^23^Na magic angle spinning (MAS, *ν_R_* =20 kHz) NMR was performed to improve the resolution and deconvolute the narrower component shown in Figure 3a and 3b. The ^23^Na MAS spectrum shows two Na_2_O_2_ resonances (purple at 9.5 and 4.5 ppm), a minor Na_2_O resonance (blue at 53 ppm), and a broader quadrupole resonance attributed to NaOH (green at 38.5 ppm), shown in Figure 3c. The isotropic shift of the attributed NaOH resonance is at a higher frequency than previous work published on NaOH (21 ppm).^[33]^ However, the quadrupole coupling constant (*C_Q_* =3.8 MHz) is similar to that of NaOH. This discrepancy in shift could be due to the lack of periodicity, as NaOH will be neighbouring Na_3_OBr, Na_2_O_2_ and Na_2_O in the mixture. The presence of NaOH is also detected by Raman as seen in Figure S5. The total percentage of impurities is 5 %, with an atomic ratio between Na_2_O_2_, Na_2_O, and NaOH of 40 : 3 : 57. This amount is substantial and yet no additional Bragg peaks were detected in the XRD patterns. Therefore, the impurities contained in the sample are likely to be poorly crystalline, which could be a result of the 10 hours of ball milling at 350 rpm carried out during the synthesis.[^34^, ^35^]

To understand how the Na environment changes with temperature, we performed temperature‐controlled static ^23^Na NMR. As shown in Figure 3d, the intensity of the Na_3_OBr peak remains constant over the sampled temperature range, although there is a reduction in the quadrupole coupling constant at elevated temperatures. This shows that the antiperovskite structure is maintained even after the transition, but the mobility of the sodium ions inside the structure increases slightly at these elevated temperatures. The variable temperature NMR also shows a significant intensity increase and peak width decrease in the narrower component on heating above 230 °C.^[36]^ These changes are typically observed for melting. Therefore, our temperature‐dependent NMR results indicate that the step increase in ionic conductivity around 250 °C is likely not an intrinsic property of Na_3_OBr.

**Figure 4:**
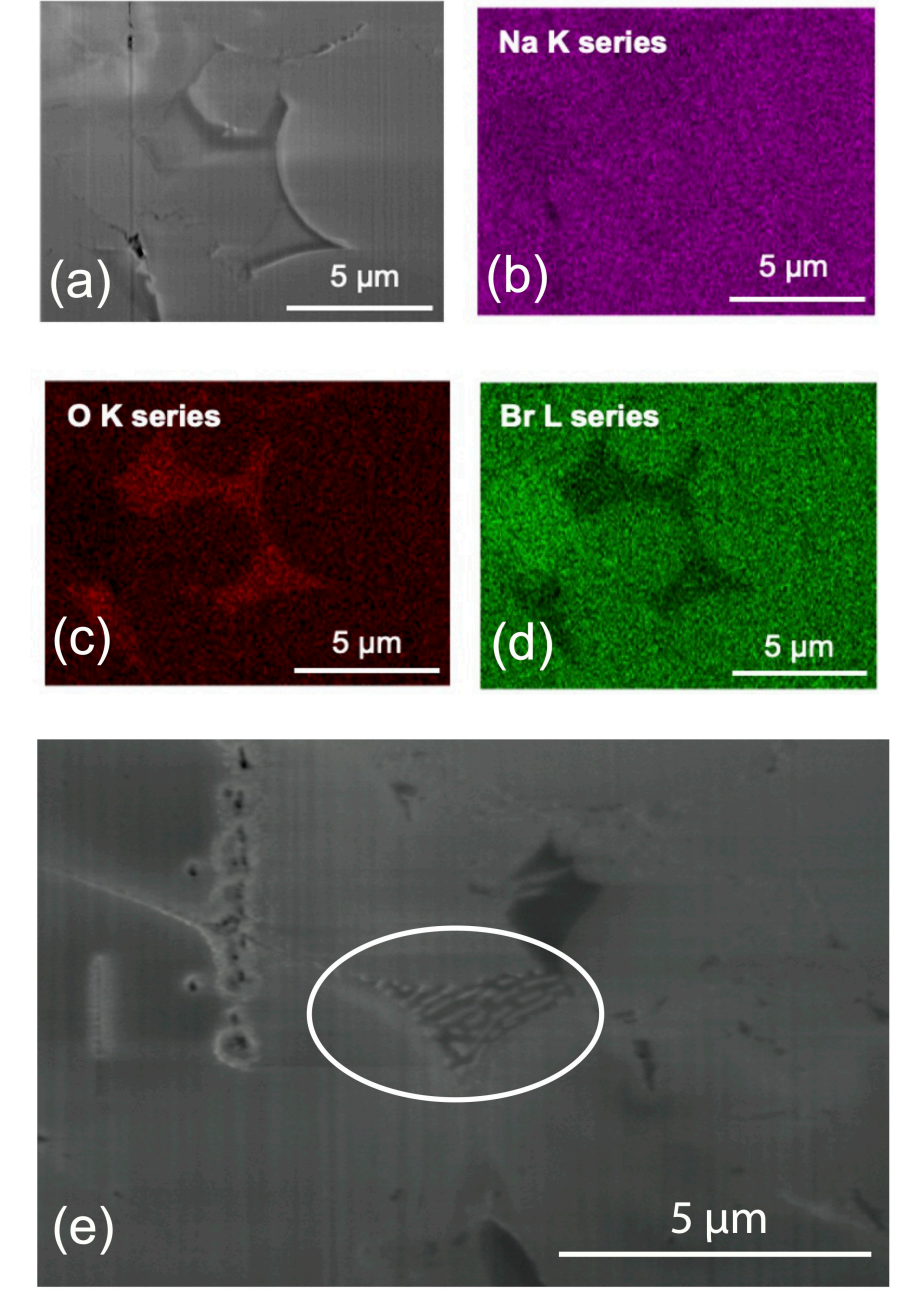
(a) Cross‐sectional secondary electron image of the Na_3_OBr pellet taken after variable temperature EIS measurements. Corresponding EDX analysis of the different elements: (b) Na (purple), (c) O (red), and (d) Br (green). Na is homogeneously distributed throughout the pellet but there are areas around some of the grain and particle boundaries which are rich in O and deficient in Br. The oxygen rich areas are not uniformly distributed throughout the pellet. (e) Cross‐sectional secondary electron image of the same pellet showing eutectic‐like lamellae between Na_3_OBr particles (circled).

Na_2_O_2_ is an impurity contained in our Na_2_O precursor. It is very hygroscopic, so it could readily react with water molecules inside the glovebox to form NaOH. Each of the impurities detected by the MAS NMR is proposed to be thermodynamically stable to above 300 °C.^[37]^ However, a eutectic between Na_2_O_2_, Na_2_O, and NaOH has been reported at ≈270 °C.^[38]^ Therefore, the endothermic peak seen in our DSC could correspond to the eutectic melting of a solid phase consisting of Na_2_O_2_, Na_2_O, and NaOH. Cross‐sectional EDX maps of our pellet after the variable temperature EIS (Figure 4) show small areas at the grain and particle boundaries that are rich in oxygen and deficient in bromine. Further, in some areas (Figure 4e), eutectic‐like lamellae are visible, which is a strong indication that an Na_2_O_2_‐Na_2_O‐NaOH eutectic mixture formed on cooling. Based on these findings, we propose that the melting of a eutectic mixture of Na_2_O_2_, Na_2_O, and NaOH is the main factor responsible for enhanced Na^+^ ionic mobility above 250 °C.

The importance of non‐bulk factors in determining the overall ionic conductivity of solid electrolytes has been previously investigated, with the majority of work focusing on the effect of grain boundaries.[^30^, ^39^, ^40^, ^41^, ^42^, ^43^] However, the influence of impurities and their associated interphase boundaries is rarely considered. This work demonstrates how small volume fractions of impurities can have a significant impact on the macroscopic properties of a solid electrolyte. While it has been established that the presence of grain boundaries is detrimental to ionic conduction in many antiperovskite electrolytes,[^39^, ^40^, ^43^] in this study the liquid phase that forms at the particle boundaries on heating above 250 °C has been found to improve the high‐temperature ionic conduction. This is supported by the Arrhenius plot of bulk and boundary ionic conductivities shown in Figure S1c and Figure S1d. The change in bulk ionic conductivity is linear with temperature, with a constant activation energy of 0.43 eV. On the contrary, there is a step change in the boundary ionic conductivity at 250 °C, the temperature at which an endothermic peak was observed in the DSC. This corresponds to a reduction in activation energy from 0.51 eV to 0.31 eV. Despite improving the high‐temperature ionic conduction, other properties of the liquid boundary phase such as an increased electronic conductivity may ultimately render this secondary phase undesirable.^[44]^

## Conclusion

We synthesised Na_3_OBr using commercially available Na_2_O and observed a non‐Arrhenius change in ionic conductivity at a temperature of about 250 °C. This coincided with an endothermic peak measured by DSC. Bulk characterisation of this material using variable temperature in situ synchrotron XRD did not show a detectable change in its crystal structure, which suggests that this behaviour does not originate from a melting or order‐disorder transition in Na_3_OBr as previously speculated. Further local characterisation using variable temperature in situ NMR revealed that the sample contained a minor fraction of poorly crystalline impurities consisting of Na_2_O, Na_2_O_2_, and NaOH. These impurities form a eutectic mixture which melts at around 250 °C, promoting Na^+^ mobility. We highlight the effects of precursor purity and the synthesis method on the final quality of the product. While the use of the same precursors and similar synthesis methods is common in prior literature, the likelihood that these lead to the formation of poorly crystalline impurity phases which affect the electrochemical performance of the material has not been understood until now. It is therefore advisable not to use commercial Na_2_O, which contains ≈20 % Na_2_O_2_, as a solid‐state synthesis precursor. Ball‐milling may reduce the crystallinity of impurity phases, which could explain why they are often overlooked in solid‐state electrolyte characterisation studies. Therefore, if a mechanochemical synthesis method is employed, it is important also to check for poorly crystalline impurities. In light of these findings, the non‐Arrhenius ionic conductivity behaviour seen in a range of other solid electrolyte materials—particularly the Li‐and K‐rich antiperovskites—should be re‐investigated. These findings will be of significant practical value to researchers attempting to optimise the synthesis of NaRAPs. Furthermore, they improve understanding of the processing‐structure‐properties relationships pertaining to NaRAPs and highlight the need to determine these relationships in other materials systems, which will accelerate the development of high‐performance solid electrolytes.

## Conflict of interest

The authors declare no conflict of interest.

1

## Supporting information

As a service to our authors and readers, this journal provides supporting information supplied by the authors. Such materials are peer reviewed and may be re‐organized for online delivery, but are not copy‐edited or typeset. Technical support issues arising from supporting information (other than missing files) should be addressed to the authors.

Supporting Information

## Data Availability

The data that support the findings of this study are available in the supplementary material of this article.
